# Supplementary material for: Shotgun Metagenomics Reveals Minor Micro“bee”omes Diversity Defining Differences between Larvae and Pupae Brood Combs
Source: Int J Mol Sci. 2024 Jan 6;25(2):741. doi: 10.3390/ijms25020741 (PMC10815634; doi:10.3390/ijms25020741)
Supplement: Supplementary file 1 [file ijms-25-00741-s001.zip › ijms-2735751-supplementary.pdf]

Supplementary material

Shotgun metagenomics reveals minor micro"*bee*"omes diversity defining differences between larvae and pupae brood combs.

Smutin Daniil, Taldaev Amir, Lebedev Egor and Adonin Leonid

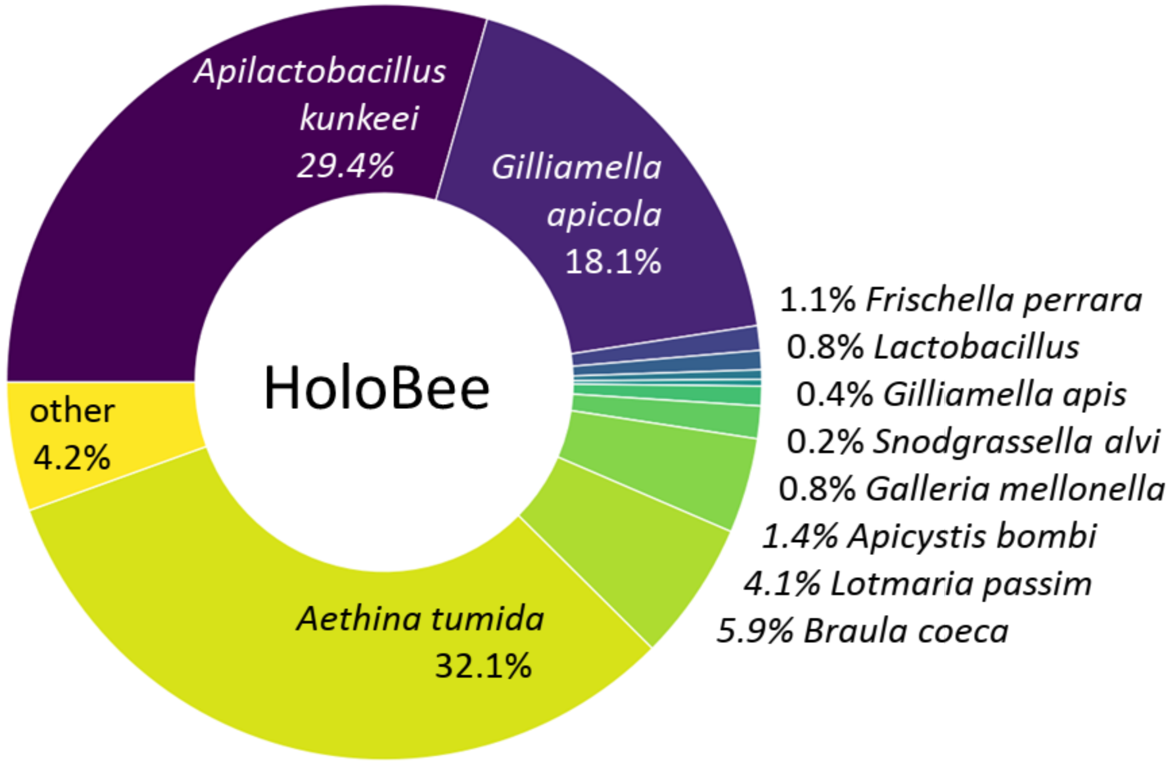

Figure S1 Classification results on the HoloBee database using Kraken 2.

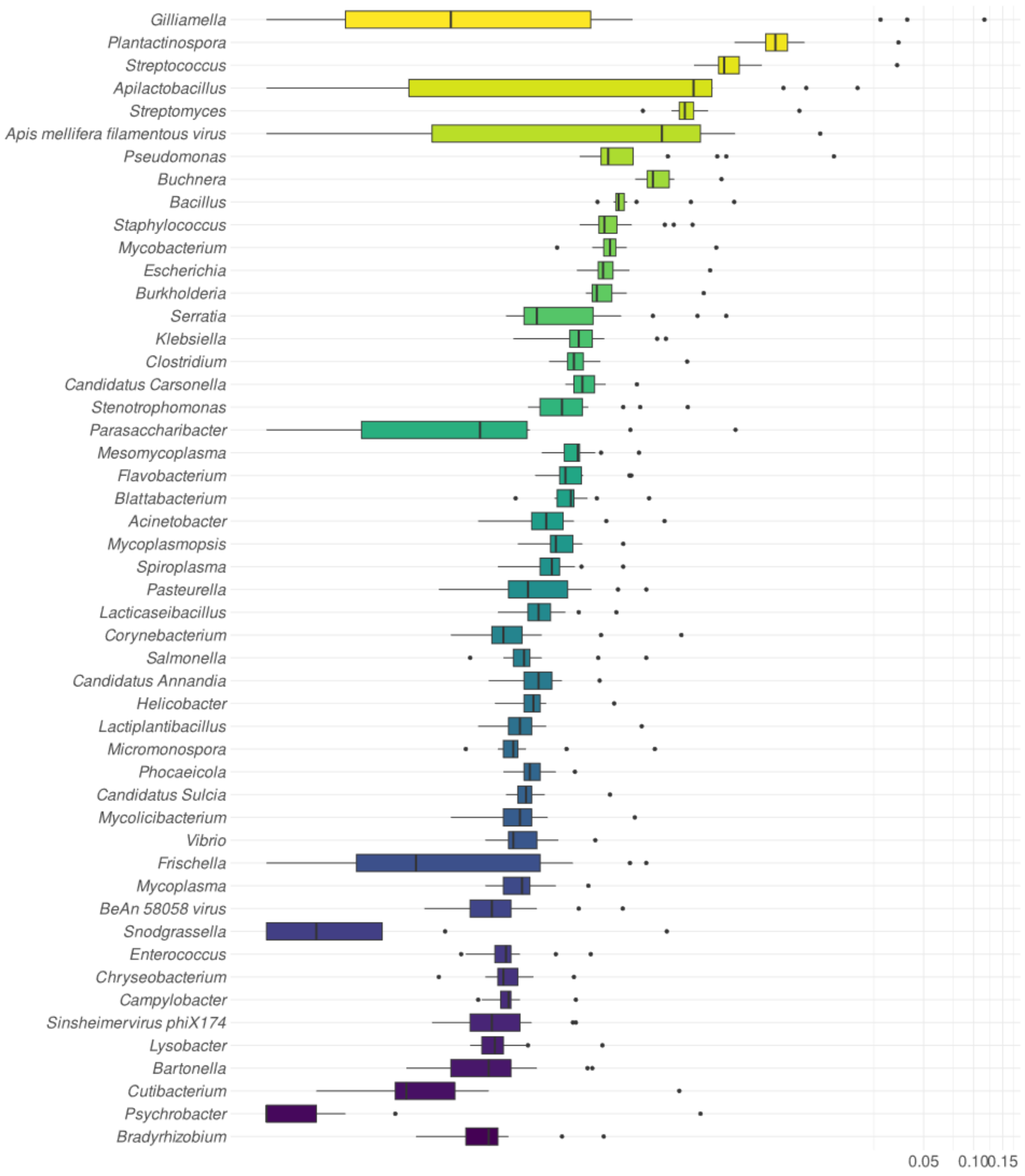

Figure S2 Box-plot diagrams of the bacterial composition of the microbiome. Dots in the diagram show outliers.

Comment S1. Comment on annotation tools classification

While the number of bacterial reads is estimated with high accuracy, this tool predicts the number of gene copies, not the real abundances [1]. Kraken 2 is known to generate a small percent of false-positives [2]. Removing false-positives and misclassified taxa from analysis is still a very complicated process because Kraken 2 finds not the exact metagenomics group but maps sequence to the tree and extract related taxa [2,3]. Tools often misclassify species [4], so we use only genera. We hypothesize that some bacteria genera still may have been misclassified. Different approaches to increase taxa classifying credibility [1,3–7] does not suit our data. Most other programs annotate only *Gilliamella apicola* reads, so evaluation using a few different approaches for Bacteria species could not be done. For numerous groups, we assume misclassification for *Buchnera* and *Plantactinosopra* as other Enterobacteriaceae, Gammaproteobacteria, and Micromonosporaceae, Actinomycota species respectively. *Buchnera aphidicola* is an obligate endosymbiont of aphids [8], but also was identified as a molecular signature in liver and other substrates metagenomes [9–11]. *Plantactinosopra* was previously detected mainly from plant microbiomes [12], while Kraken 2 sometimes characterizes microbial signatures from different sources in this way [9,13–17].

Kaiju classified only 22000 reads on the RefSeq database. 97% of that diversity belongs to Bacteria, and other is almost only unclassified viruses. More than 78% of the classification is made up of *Gilliamella apicola* reads. But it classifies more than 8000 reads on a custom fungal database, whereas Kraken 2 predicts only few taxonomic units.

All samples were infected by *Varroa destructor* and some also by *Apis mellifera filamentosus virus* (*AMFV*), which was confirmed by Kraken 2 and Kaiju both on similar levels.

The abundance of none of the classified species ever exceeds 5% of the total fungal diversity. The total content of classified reads of fungi is almost at the same level, especially if *Gilliamella* is excluded from the classification (Figure S3). On the one hand, it is an indicator of fungal diversity consistency. On the other hand, most variance in both classified and bacteria amounts refer to *Gilliamella* level.

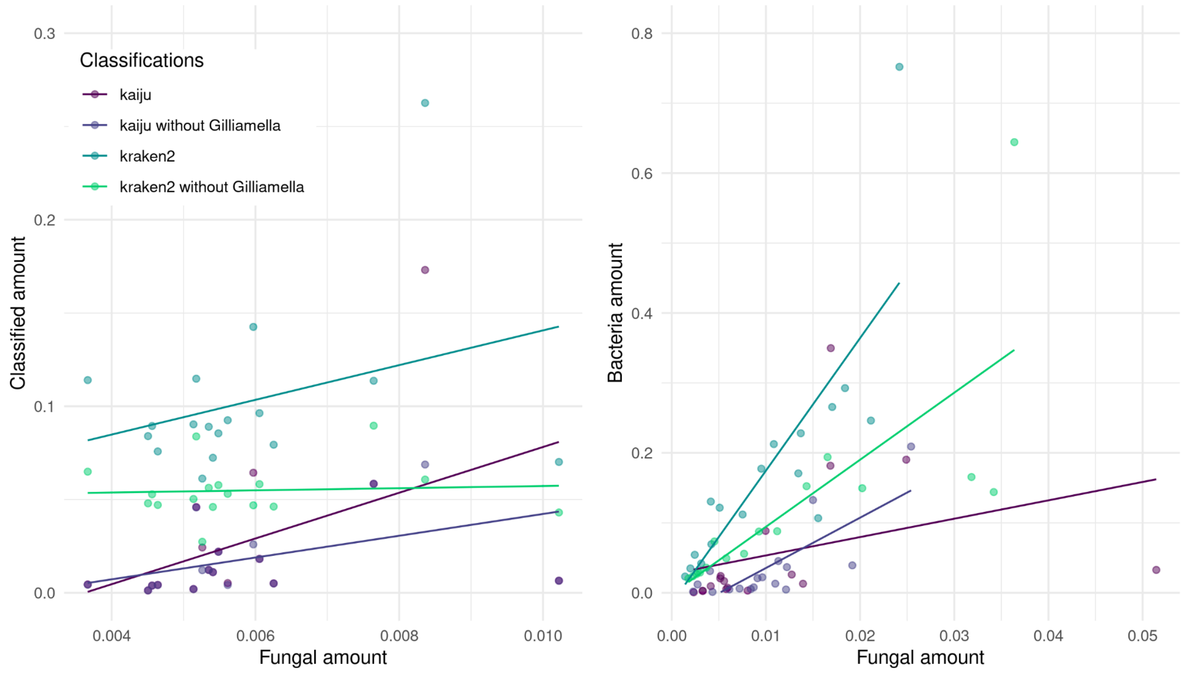

Figure S3. Relationship between the number of all classified (left) and bacterial (right) reads and the number of classified fungal reads. Each dot shows a different sample.

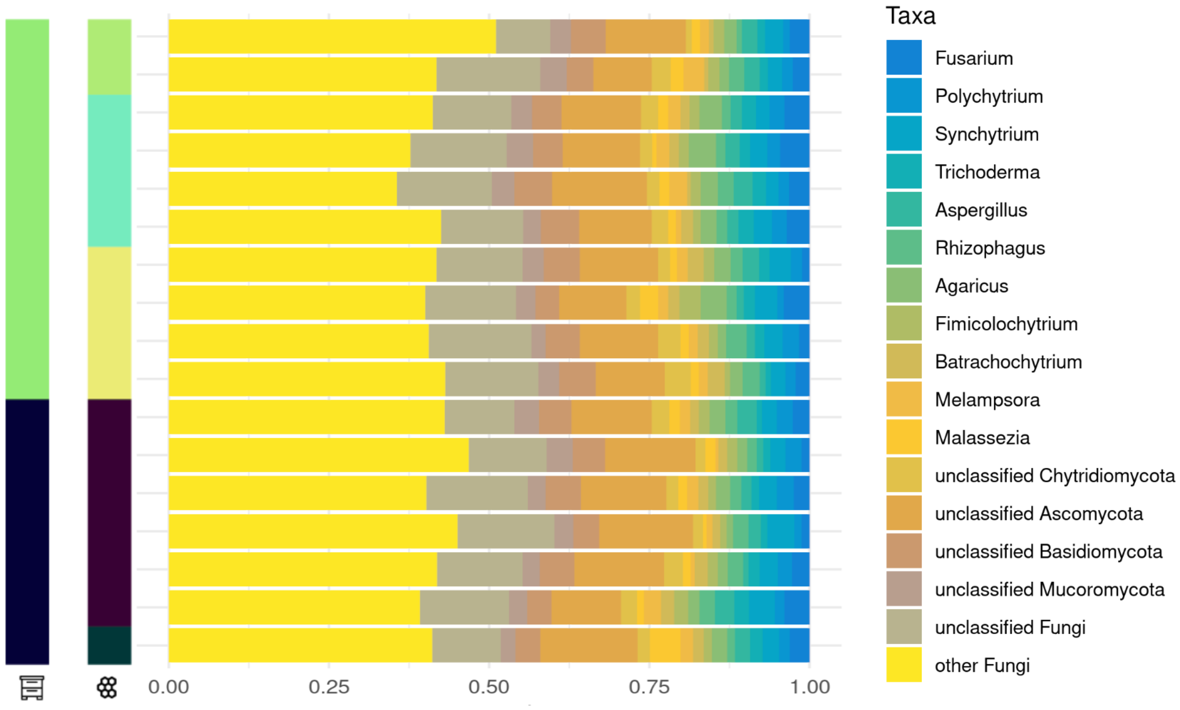

Figure S4. Composition of fungal sequences in the microbiome based on Kaiju results on Fungi database. The side colors indicate the hive and sampling frame, while the column sizes correspond to the percentage of all reads.

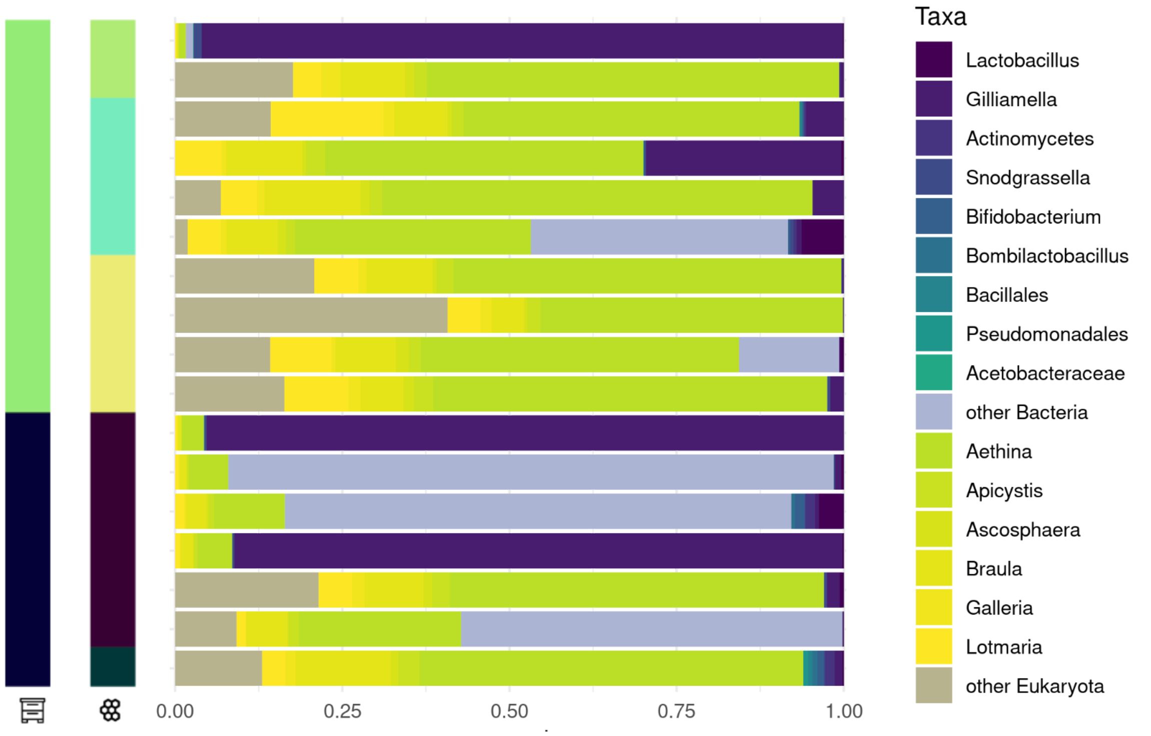

Figure S5. Composition of organism sequences in the microbiome based on Kraken 2 results on HoloBee database. The side colors indicate the hive and sampling frame, while the column sizes correspond to the percentage of all reads.

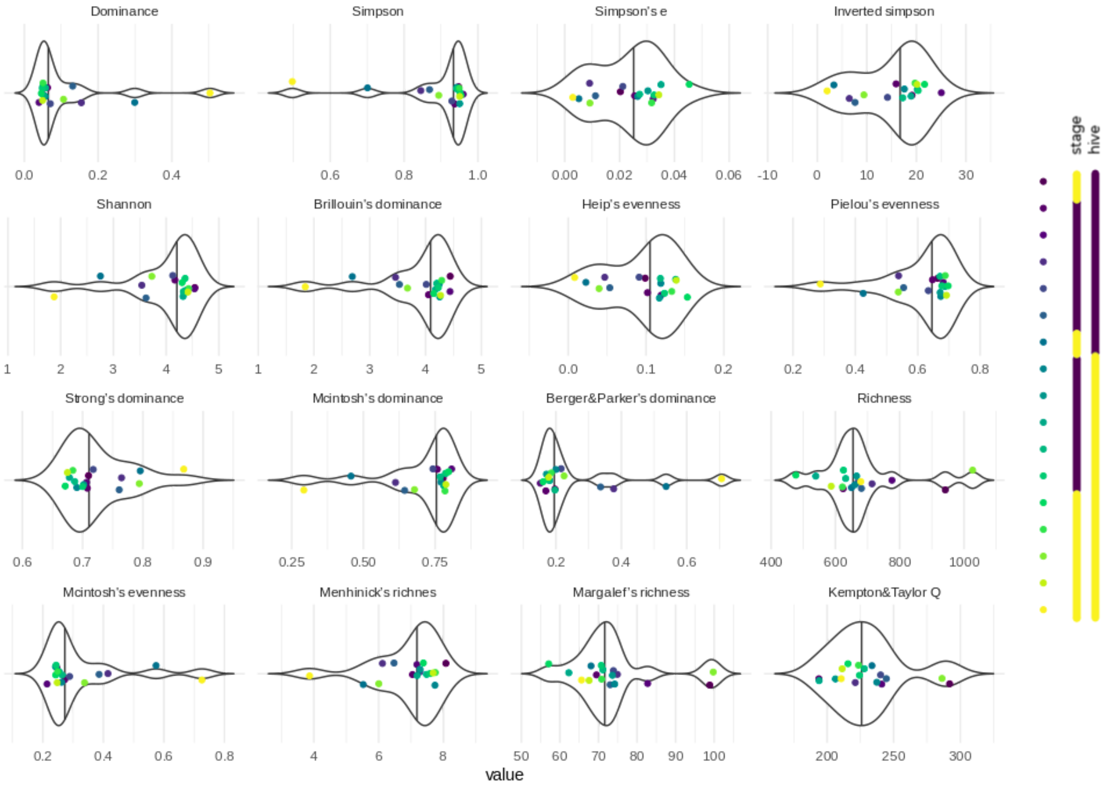

Figure S6. Alpha diversity measures on a full dataset based on genus classifications. Threshold for taxa amount used in analysis was 10^{-5}. Legend: stage, purple - larvae, yellow - pupa; hive, 1st - purple, 2nd - yellow.

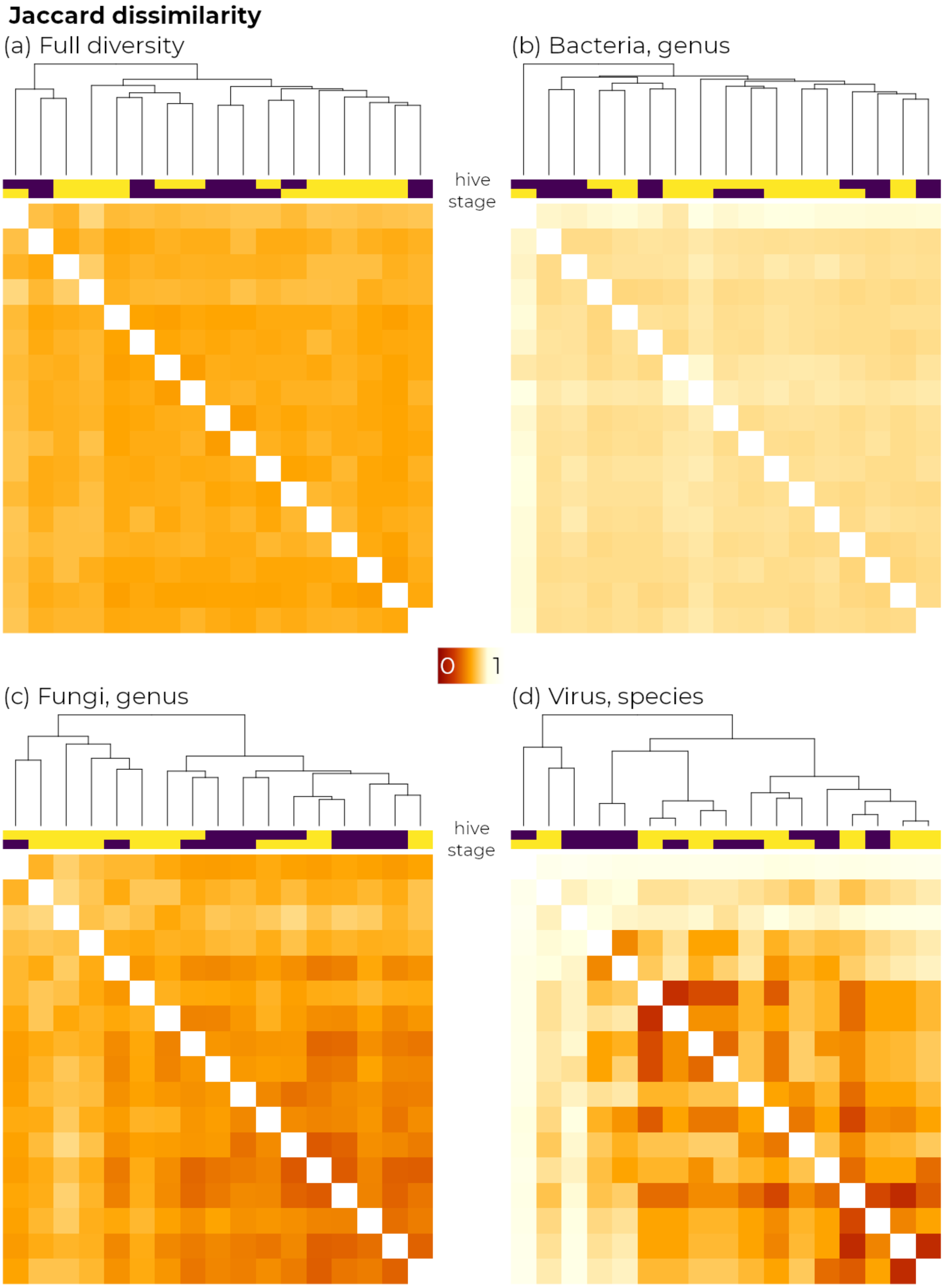

Figure S7. Beta diversity Jaccard measures on different dataset parts. Full dataset has been assembled from all other subsets - Fungi and Bacteria genus and Virus species annotations. Threshold for taxa amount used in analysis was 10^{-5}. Legend: stage, purple - larvae, yellow - pupa; hive, 1st - purple, 2nd - yellow.

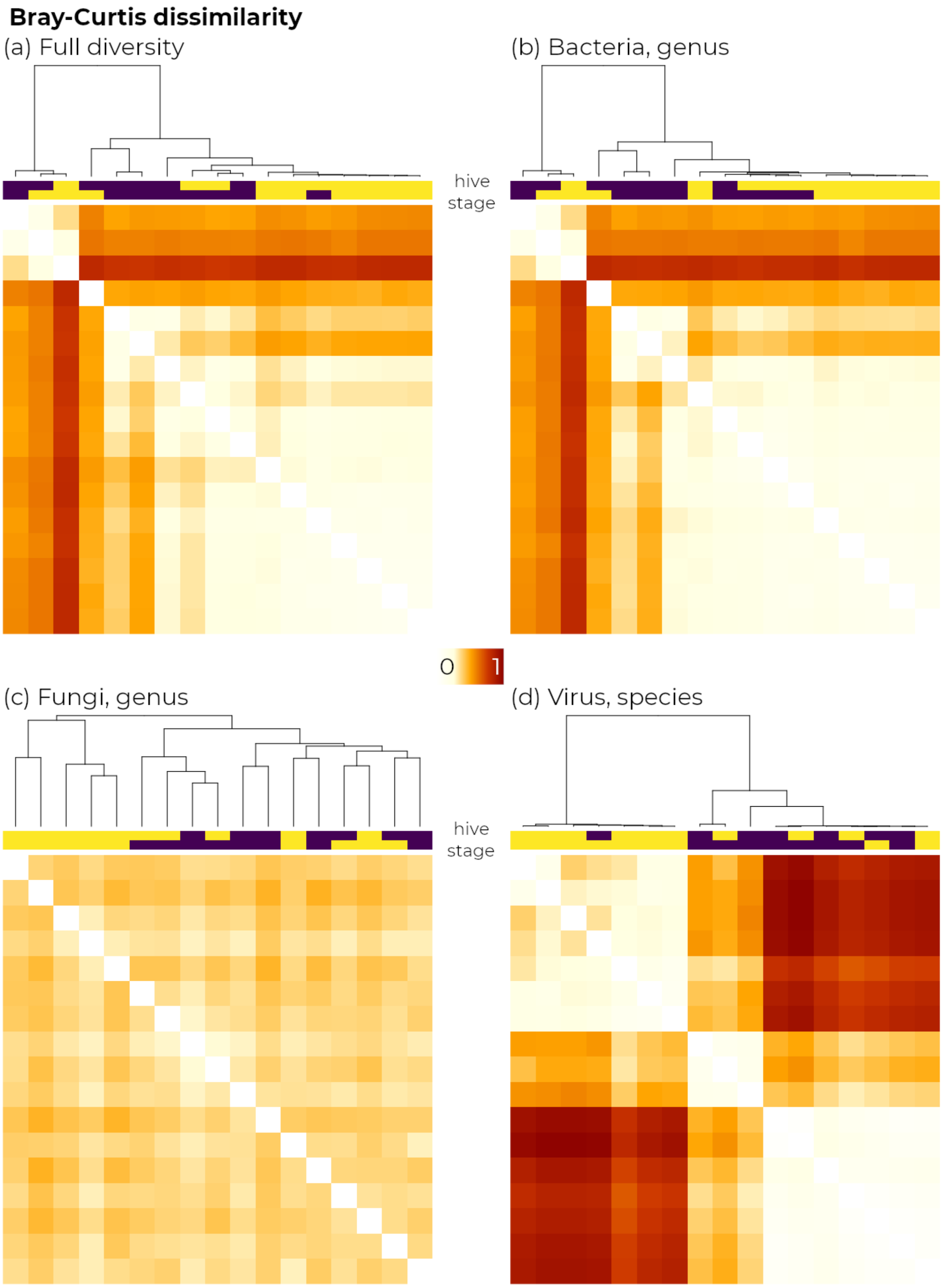

Figure S8. Beta diversity Bray-Curtis measures on different dataset parts. Full dataset has been assembled from all other subsets - Fungi and Bacteria genus and Virus species annotations. Threshold for taxa amount used in analysis was 10^{-5}. Legend: stage, purple - larvae, yellow - pupa; hive, 1st - purple, 2nd - yellow.

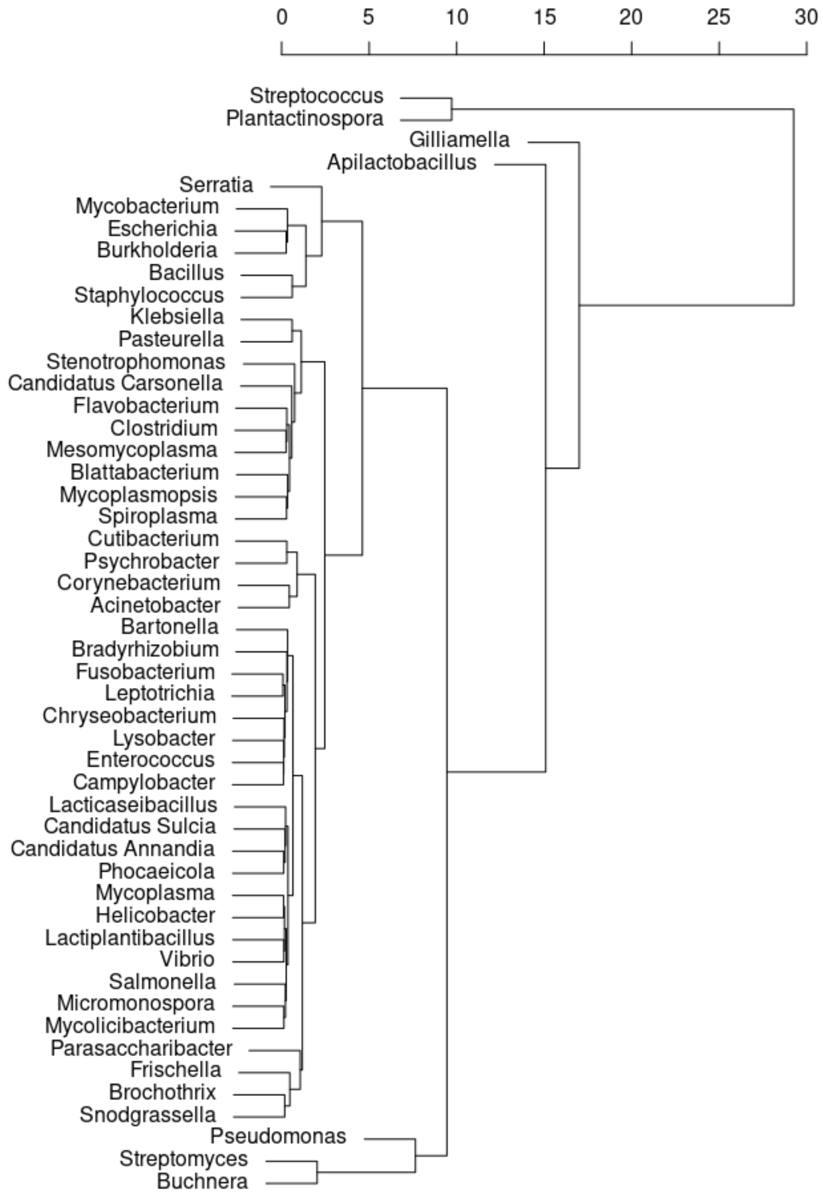

Figure S9. Bacteria clustering results using ward.D2 clustering method and euclidean distance.

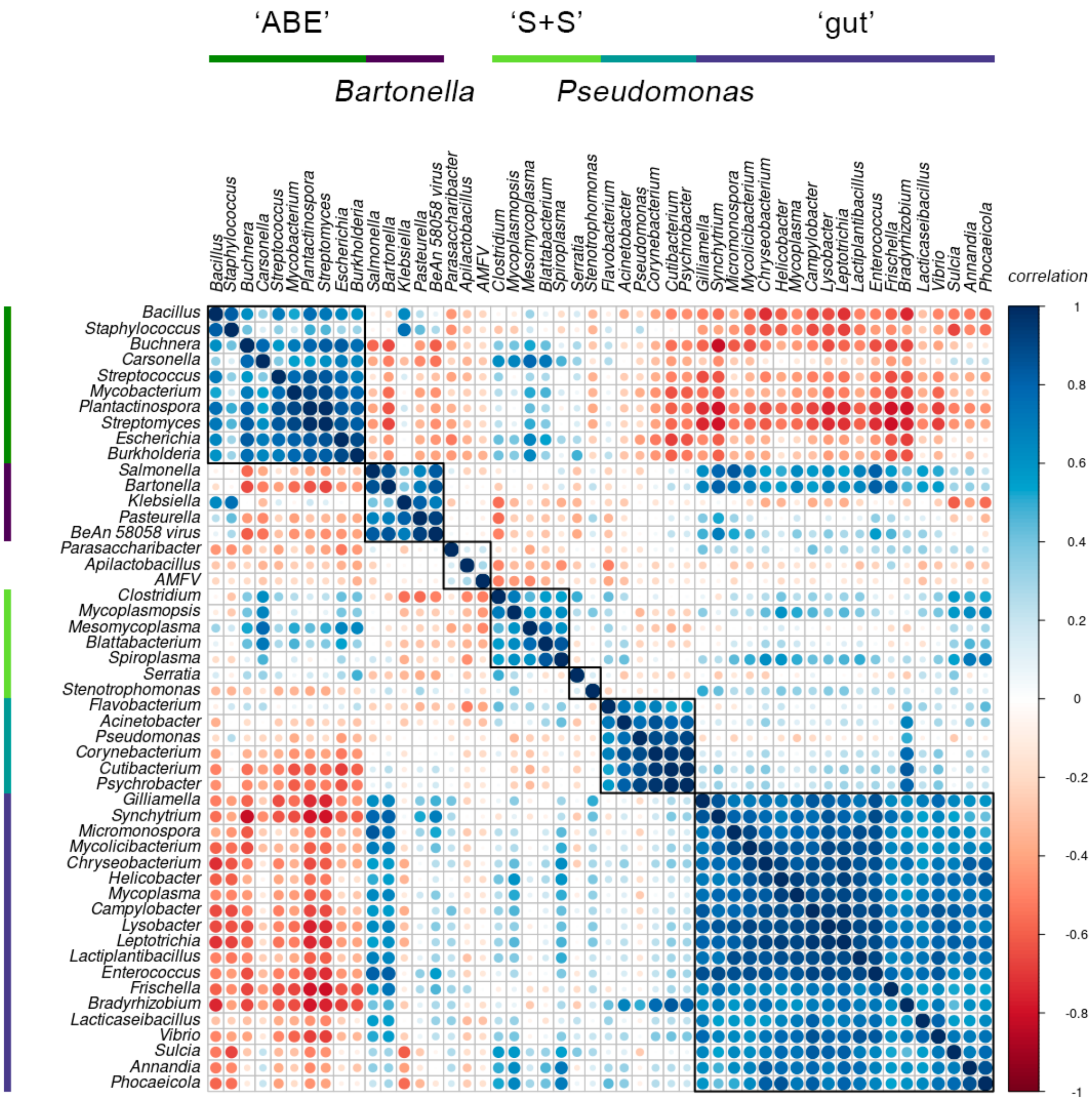

Figure S10. Correlation of top-50 Bacteria taxa by log2 number of reads in samples. Size and color represent correlation levels. Clustering between taxa performed using euclidean distance and complete method.

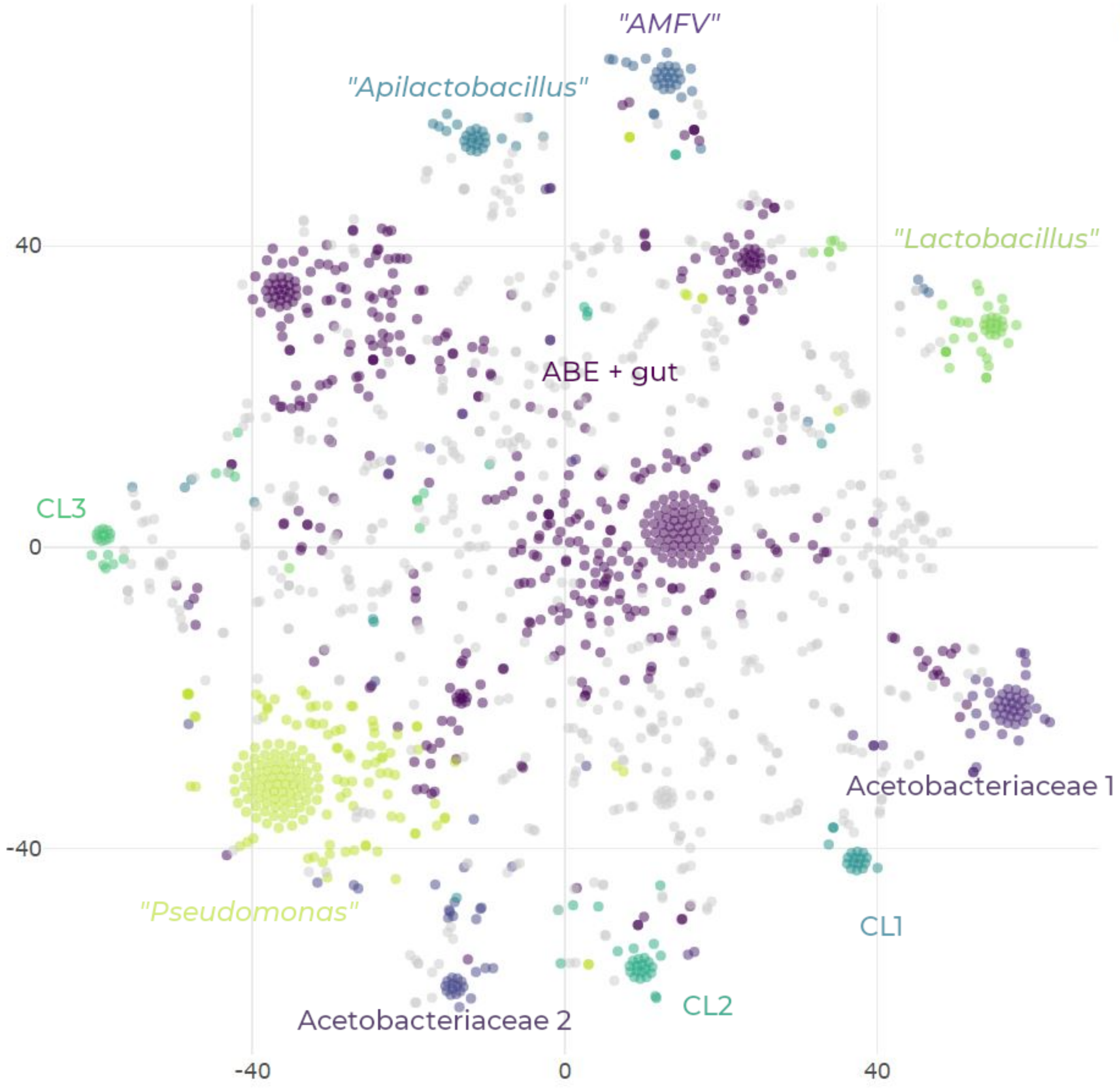

Figure S11. tSNE of the most represented genera. Full dataset has been assembled from all other subsets - Fungi and Bacteria genus and Virus species annotations. Number of generations is 1000. Text labels for clusters (see description in text).

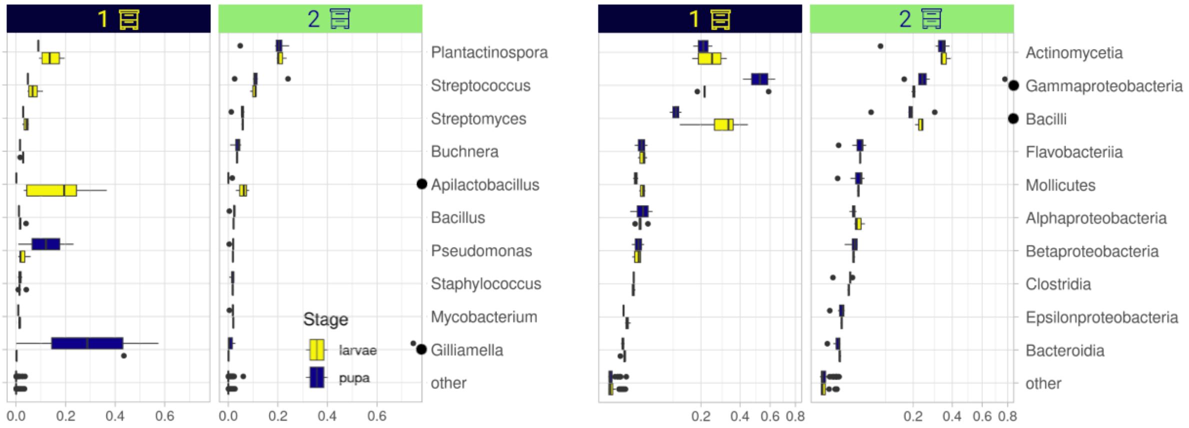

Figure S12. Box-plot diagrams of microbiome compositions. Dots in the diagram show outliers. Taxa whose number of representatives reliably differs between stages are marked with circles.

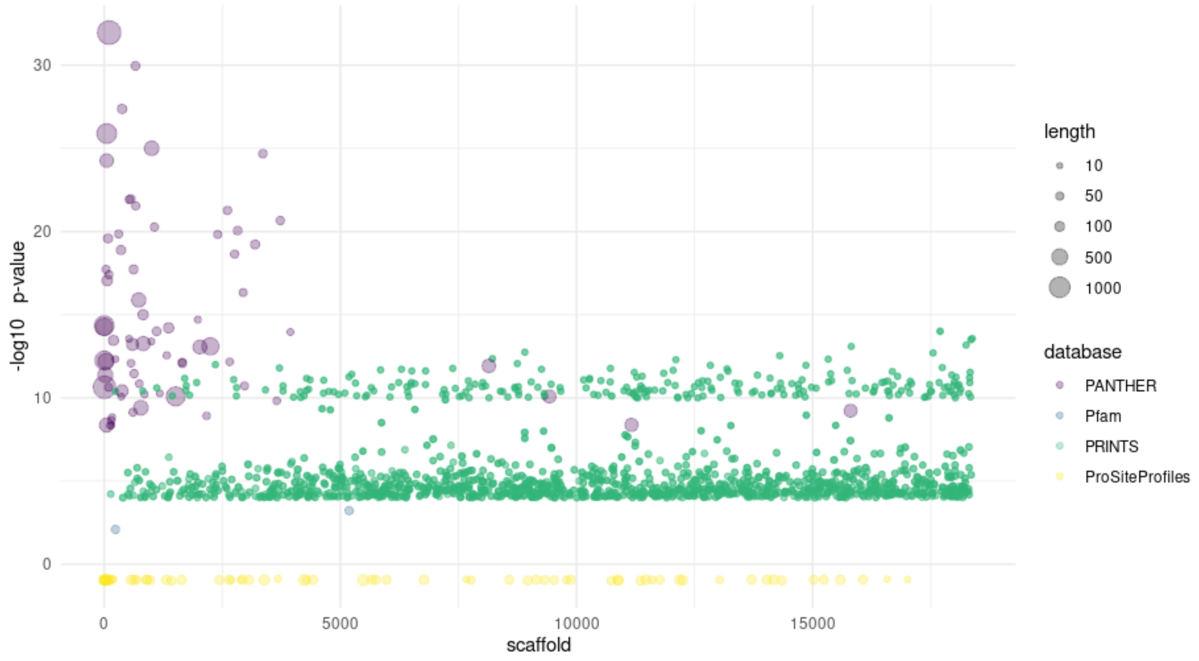

Figure S13. Functional analysis distribution results obtained using InterProScan across 4 databases. The y-axis indicates automatically generated p-value of classification. On the x-axis is a number of scaffolds. Scaffolds were sorted based on their length. Test was performed on the IDBA-UD pooled assembly. Genes were annotated with prodigal.

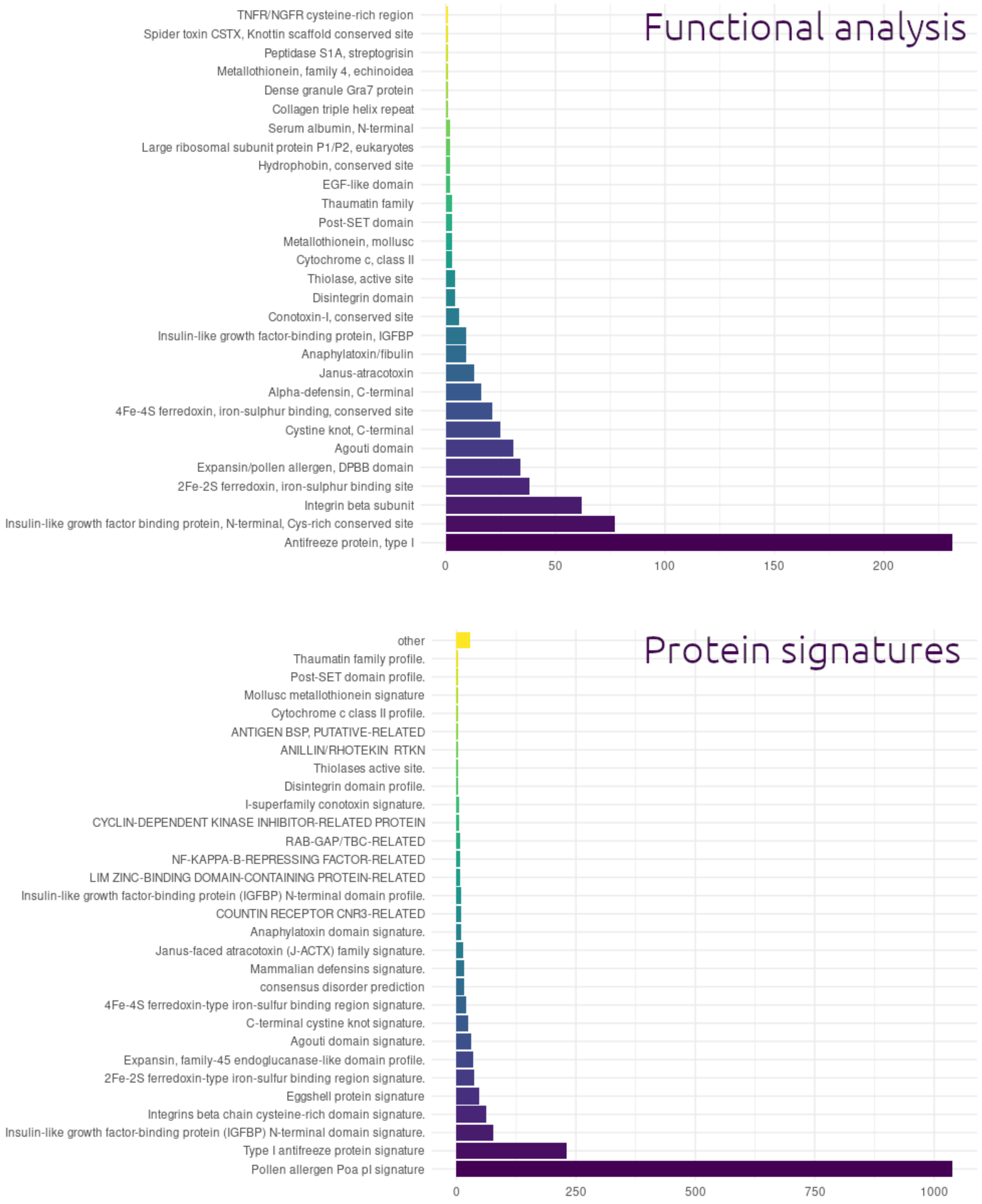

Figure S14. Functional analysis (top) and results protein signatures (bottom) generated using InterProScan. The x-axis indicates the number of genes from the IDBA-UD pooled assembly. Genes were annotated with prodigal.

References:

[1] Sun Z, Huang S, Zhang M, Zhu Q, Haiminen N, Carrieri AP, et al. Challenges in Benchmarking Metagenomic Profilers. Nat Methods 2021;18:618–26. https://doi.org/10.1038/s41592-021-01141-3.

[2] Wood DE, Lu J, Langmead B. Improved metagenomic analysis with Kraken 2. Genome Biol 2019;20:257. https://doi.org/10.1186/s13059-019-1891-0.

[3] Lu J, Rincon N, Wood DE, Breitwieser F, Pockrandt C, Langmead B, et al. Metagenome analysis using the Kraken software suite. Nat Protoc 2022;17:2815–39. https://doi.org/10.1038/s41596-022-00738-y.

[4] Johnson J, Sun S, Fodor AA. Systematic classification error profoundly impacts inference in high-depth Whole Genome Shotgun Sequencing datasets 2022:2022.04.04.487034. https://doi.org/10.1101/2022.04.04.487034.

[5] Garrido-Sanz L, Senar MÀ, Piñol J. Drastic reduction of false positive species in samples of insects by intersecting the default output of two popular metagenomic classifiers. PLOS ONE 2022;17:e0275790. https://doi.org/10.1371/journal.pone.0275790.

[6] Ye SH, Siddle KJ, Park DJ, Sabeti PC. Benchmarking Metagenomics Tools for Taxonomic Classification. Cell 2019;178:779–94. https://doi.org/10.1016/j.cell.2019.07.010.

[7] Kang DD, Li F, Kirton E, Thomas A, Egan R, An H, et al. MetaBAT 2: an adaptive binning algorithm for robust and efficient genome reconstruction from metagenome assemblies. PeerJ 2019;7:e7359. https://doi.org/10.7717/peerj.7359.

[8] Baumann P, Baumann L, Lai CY, Rouhbakhsh D, Moran NA, Clark MA. Genetics, physiology, and evolutionary relationships of the genus Buchnera: intracellular symbionts of aphids. Annu Rev Microbiol 1995;49:55–94. https://doi.org/10.1146/annurev.mi.49.100195.000415.

[9] Li W, Larsen A, Murphy B, Fregulia P. Liver microbial community and associated host transcriptome in calves with feed induced acidosis. Front Vet Sci 2023;10.

[10] Martyn C, Hayes B, Lauko D, Mithun E, Castañeda G, Bosco-Lauth A, et al. mNGS Investigation of Single Ixodes pacificus Ticks Reveals Diverse Microbes, Viruses, and a Novel mRNA-like Endogenous Viral Elements. 2022. https://doi.org/10.1101/2022.08.17.504163.

[11] Mehta P, Soni J, Chattopadhyay P, Devi P, Habyarimana T, Tardalkar K, et al. Single-cell RNA-Seq reveals intracellular microbial diversity within Immune cells during SARS-CoV-2 Infection and Recovery. iScience 2023:108357. https://doi.org/10.1016/j.isci.2023.108357.

[12] Contreras-Castro L, Maldonado LA, Quintana ET, Raggi L, Sánchez-Flores A. Draft Genome Sequence of Two Marine Plantactinospora spp. from the Gulf of California. Genome Announc 2018;6:e00436-18. https://doi.org/10.1128/genomeA.00436-18.

[13] Sarmiento-Ramírez JM, van der Voort M, Raaijmakers JM, Diéguez-Uribeondo J. Unravelling the microbiome of eggs of the endangered sea turtle Eretmochelys imbricata identifies bacteria with activity against the emerging pathogen Fusarium falciforme. PloS One 2014;9:e95206. https://doi.org/10.1371/journal.pone.0095206.

[14] Pascar J. Characterizing microbiome variation in wild populations of mosquito species. Theses - ALL 2022.

[15] Usié A, Leão C, Gaspar D, Monteiro H, Tábuas L, Bettencourt E, et al. A metagenomics approach to characterize the footrot microbiome in Merino sheep. Vet Microbiol 2023;281:109745. https://doi.org/10.1016/j.vetmic.2023.109745.

[16] Kim D, Yu Y, Jung KS, Kim YH, Kim J-J. Tumor Microenvironment Can Predict Chemotherapy Response of Patients with Triple-Negative Breast Cancer Receiving Neoadjuvant Chemotherapy. Cancer Res Treat 2023. https://doi.org/10.4143/crt.2023.330.

[17] Carstens CK, Salazar JK, Sharma SV, Chan W, Darkoh C. Evaluation of the kitchen microbiome and food safety behaviors of predominantly low-income families. Front Microbiol 2022;13:987925. https://doi.org/10.3389/fmicb.2022.987925.
